# The Kinase Function of MSK1 Regulates BDNF Signaling to CREB and Basal Synaptic Transmission, But Is Not Required for Hippocampal Long-Term Potentiation or Spatial Memory

**DOI:** 10.1523/ENEURO.0212-16.2017

**Published:** 2017-02-20

**Authors:** Stephanie Daumas, Christopher J. Hunter, Rajen B. Mistry, Lorenzo Morè, Lucia Privitera, Daniel D. Cooper, Kathleen M. Reyskens, Harry T. Flynn, Richard G. M. Morris, J. Simon C. Arthur, Bruno G. Frenguelli

**Affiliations:** 1 Centre for Cognitive and Neural Systems, The University of Edinburgh, Edinburgh EH8 9JZ, United Kingdom; 2 MRC Protein Phosphorylation Unit, College of Life Sciences, The University of Dundee, Dundee DD1 5EH, United Kingdom; 3 School of Life Sciences, The University of Warwick, Coventry CV4 7AL, United Kingdom; 4 Division of Cell Signalling and Immunology, College of Life Sciences, The University of Dundee, Dundee DD1 5EH, United Kingdom

**Keywords:** BDNF, CREB, learning, LTP, memory, MSK1

## Abstract

The later stages of long-term potentiation (LTP) *in vitro* and spatial memory *in vivo* are believed to depend upon gene transcription. Accordingly, considerable attempts have been made to identify both the mechanisms by which transcription is regulated and indeed the gene products themselves. Previous studies have shown that deletion of one regulator of transcription, the mitogen- and stress-activated kinase 1 (MSK1), causes an impairment of spatial memory. Given the ability of MSK1 to regulate gene expression via the phosphorylation of cAMP response element binding protein (CREB) at serine 133 (S133), MSK1 is a plausible candidate as a prime regulator of transcription underpinning synaptic plasticity and learning and memory. Indeed, prior work has revealed the necessity for MSK1 in homeostatic and experience-dependent synaptic plasticity. However, using a knock-in kinase-dead mouse mutant of MSK1, the current study demonstrates that, while the kinase function of MSK1 is important in regulating the phosphorylation of CREB at S133 and basal synaptic transmission in hippocampal area CA1, it is not required for metabotropic glutamate receptor-dependent long-term depression (mGluR-LTD), two forms of LTP or several forms of spatial learning in the watermaze. These data indicate that other functions of MSK1, such as a structural role for the whole enzyme, may explain previous observations of a role for MSK1 in learning and memory.

## Significance Statement

The nuclear kinase mitogen- and stress-activated kinase 1 (MSK1) has been identified as a possible link between cell-surface neurotransmitter receptors and the gene expression necessary for long-term memory: by coupling the activation of BDNF receptors to the regulation of transcription via the phosphorylation of cAMP response element binding protein (CREB), MSK1 unites a neurotrophin heavily implicated in synaptic plasticity with changes in gene expression. Using a kinase-dead MSK1 mouse mutant, we show that, while MSK1 is necessary for CREB phosphorylation and the regulation of basal synaptic transmission, it is not required for metabotropic glutamate receptor-dependent long-term depression (mGluR-LTD), long-term potentiation (LTP), or several forms of spatial reference memory (SRM). MSK1 may instead play a homeostatic role in the CNS that allows synapses to adapt to prevailing synaptic or sensory experience.

## Introduction

The intracellular mechanisms that link the activation of cell surface neurotransmitter receptors to the genomic changes promoting neuronal morphological and functional adaptations has been a topic of considerable interest. This interest has arisen from the potential to both identify signaling pathways responsible for these changes, and to use this knowledge to intervene pharmacologically in a host of developmental, neurological, and psychiatric conditions (Guzman-Karlsson et al., 2014; Lynch et al., 2014).

In this regard, the nuclear kinase mitogen- and stress-activated kinase (MSK) (Deak et al., 1998) is well placed to couple activity at the cell surface to changes in gene expression. Of the two isoforms of MSK (MSK1 and MSK2), MSK1 is most highly expressed in brain (Arthur et al., 2004) and is activated in response to neurotrophins, including brain-derived neurotrophic factor (BDNF) (Arthur, 2008; Frenguelli and Corrêa, 2012; Reyskens and Arthur, 2016). The stimulation of BDNF TrkB receptors results in the activation of a number of signaling pathways including the MAPK cascade, and in particular of ERK1/2 and p38 (Minichiello, 2009; Panja and Bramham, 2014). These enzymes translocate to the nucleus and are responsible for the direct activation of MSK1 via phosphorylation of key residues on the C-terminal kinase domain, which then phosphorylates residues on the N-terminal kinase domain (McCoy et al., 2005). Once activated, the N-terminal kinase domain of MSK1 phosphorylates cAMP response element binding protein (CREB) at serine 133 (S133) (Deak et al., 1998; Arthur, 2008; Frenguelli and Corrêa, 2012; Reyskens and Arthur, 2016). From observations in *Aplysia* (Dash et al., 1990), *Drosophila* (Yin et al., 1994), and mice (Bourtchuladze et al., 1994), CREB has emerged as an evolutionarily-conserved mechanism by which neurons convert activity into persistent modifications of synaptic function and the formation and stabilization of memories (Sakamoto et al., 2011; Kandel, 2012; Kida and Serita, 2014).

Accordingly, by linking BDNF to CREB-dependent gene transcription, MSK1 is positioned to respond to the many experiential and synaptic stimuli that provoke the release of BDNF and to convert these stimuli into long-term structural, functional, and cognitive adaptations. Indeed, prior work using MSK single or double knock-out mice has suggested that MSKs regulate neurogenesis (Choi et al., 2012; Karelina et al., 2012; Karelina et al., 2015) and BDNF-induced CREB phosphorylation (Arthur et al., 2004). These observations, as well as a role for MSK1 in posttranslational modifications of histones (Chwang et al., 2007; Chandramohan et al., 2008), and in the regulation of the plasticity-related protein Arc/Arg3.1 (Shepherd and Bear, 2011; Corrêa et al., 2012), may explain the reported deficits in MSK1 knock-out mice in spatial learning in the watermaze; fear conditioning (Chwang et al., 2007); the display of behavioral immobility in the forced swim test (Chandramohan et al., 2008); learning in the Barnes maze; and discrimination in novel object recognition (Karelina et al., 2012).

Although these knock-out studies suggest an important role of MSK1 in various aspects of learning and memory, this role may involve other aspects of MSK1 beyond its kinase function. For example, it has been reported that MSK1 forms a structural complex with ERK1/2 and the glucocorticoid receptor which is necessary for transcription of the immediate early genes c-Fos and Egr-1 (Gutierrez-Mecinas et al., 2011). Thus, the phenotype of knock-outs of MSK1 does not directly discriminate between structural and kinase roles for MSK1 in synaptic and cognitive function. In order to address this issue, an MSK1 mutant has been generated in which the kinase activity of MSK1 is selectively inactivated, but one in which protein levels of MSK1 remain close to wild-type levels (Corrêa et al., 2012). This kinase-dead mutation involves the knock-in substitution of an alanine for the aspartate at position 194 in the DFG motif of the N-terminal kinase domain of the endogenous MSK1 gene. Mutations in the DFG motif are commonly used to abolish the kinase activity of enzymes (Moran et al., 1988; Vijayan et al., 2015), and confirmation that this results in a kinase-dead version of MSK1 (MSK1 KD) was previously evidenced by the inability of MSK1 KD to phosphorylate peptide substrates, even when overexpressed in cell lines (Deak et al., 1998; McCoy et al., 2005; Corrêa et al., 2012).

Using this MSK1 KD mutant, we show that, while there is reduction in BDNF stimulation of CREB phosphorylation and a deficit in basal synaptic transmission in hippocampal area CA1, there is no change in paired-pulse facilitation, metabotropic glutamate receptor-dependent long-term depression (mGluR-LTD), or tetanus- or theta-burst-induced long-term potentiation (LTP). Moreover, we observe no overt deficits in various watermaze paradigms. These observations suggest that, under conditions of typical rearing of experimental rodents, the kinase activity of MSK1 is not necessary for several forms of synaptic plasticity and spatial learning, but may instead be required for homeostatic adaptation to prevailing synaptic activity.

## Materials and Methods

### Animals

A kinase-dead MSK1 mouse was generated by mutating Asp194 to Ala (D194A) in the DFG motif in the endogenous MSK1 gene (Corrêa et al., 2012). The knock-in was produced by TaconicArtemis GmbH using standard targeting methods in C57BL/6 ES cells. Routine genotyping was carried out by PCR using the primers 5’-CGGCCATGTGGTGCTGACAGC-3’ and 5’-GGGTCAGAGGCCTGCACTAGG-3’, which gives 378- and 529-bp products for wild-type and targeted alleles, respectively. Western blottings of MSK1 protein (n = 4 per genotype) revealed that MSK1 was expressed in MSK1 KD mice at approximately 65% of the levels found in wild-type mice (data not shown).

Unless otherwise stated, all mice were kept in individually ventilated cages (Tecniplast Blue Line 1284L) with a sawdust substrate, paper shavings and at least one cardboard play tunnel with water and food provided *ad libitum*. Mice were kept in social groups where possible with a maximum of five mice per cage and maintained on a 12/12 h light/dark cycle with lights on at 7 A.M. The care and accommodation of all the animals held in the facility comply with the standards set out in relevant codes of practice for the housing and care of animals used for scientific purposes.

#### Extracellular recordings

Male C57BL/6 WT and MSK1 KD mice (two to five months old) were killed by cervical dislocation in accordance with appropriate animal welfare legislation. Sagittal brain slices (400 µm) were prepared, and extracellular recordings were made from stratum radiatum in area CA1 at 32-33°C from slices that were either submerged in aCSF or, in the case of the Actinomycin-D (Act-D) experiments, held at a humidified and oxygenated air/aCSF interface. A two-pathway stimulation protocol was adopted with each electrode placed either side of the recording electrode. Each pathway was stimulated in an alternating fashion with either 60 s (submerged slices) or 90 s (interface slices) between stimuli to a given pathway. A prolonged interval between stimuli avoids activity-dependent fatigue of LTP (Fonseca et al., 2006a; Villers et al., 2012). That the stimulated fiber pathways were convergent but independent was confirmed with a cross-pathway paired-pulse facilitation protocol (50-ms interpulse interval) that showed no facilitation across pathways.

Stimulus input/output curves were generated over a range of electrical stimuli from 10-300 µA (0.1 ms in duration) using a Digitimer DS3 constant current isolated stimulator. Paired-pulse facilitation was measured over a range of 50 to 350 ms interpulse interval.

LTP was induced using two protocols: a tetanus of 100 stimuli at 100 Hz, and a theta-burst stimulation (TBS) protocol at test intensity (1 mV fEPSP amplitude in submerged slices; 3 mV fEPSP amplitude in interface slices). Bursts consisted of four stimuli at 100 Hz. Each train was composed of 10 bursts separated by 200 ms. Trains were repeated three times with an interval of 20 s. That TBS resulted in transcription-dependent LTP was tested in two series of experiments (in interface chambers) using either 40 µM Act-D (in 0.08% DMSO) applied for the duration of the experiment (>30 min before TBS and at least 80 min after TBS), or 25 µM Act-D (0.05% DMSO) given 15 min before and until 15 min after TBS. An additional series of interleaved experiments were performed in DMSO, which was present throughout the experiments at the level (0.08%) found in the 40 µM Act-D experiments.

mGluR-LTD was induced in hippocampal slices via the application of the Group I (GI) mGluR agonist DHPG (100 µM; 10 min) in the presence of both the GABA_A_ receptor antagonist picrotoxin (50 µM) and the NMDA receptor glycine site antagonist L689,560 (5 µM).

Stimulation and recording parameters, as well as the analysis of evoked fEPSPs, were under the control of WinLTP data acquisition software. Experiments were interleaved and performed blind to the genotype of the mice, which was revealed only after the experiments had been analysed and confirmed with *post hoc* genotyping.

### Immunohistochemistry for CREB phosphorylation at S133

After cutting, hippocampal slices (300 µm) were suspended on a mesh located within a 50 ml beaker (up to four slices/beaker) for 3 h in oxygenated circulating aCSF at 34°C. Slices were then either treated with 50 ng/ml recombinant human BDNF (Cell Guidance Systems; GFH1) or forskolin (50 µM; Sigma Aldrich; F6886) for 10 min or left untreated to serve as parallel time controls. Treated and untreated slices were rapidly immersed in 4 % paraformaldehyde in PBS, pH 7.4, and fixed overnight. The slices were washed three times in PBS and then incubated at room temperature in a solution containing both 10 % goat serum, to prevent nonspecific antibody binding, and 0.4 % Triton X-100 in PBS to permeabilize slices. After three washes in PBS the slices were incubated for 4 h at room temperature with a phospho-CREB primary antibody targeting S133 (rabbit mAb; Cell Signalling; #9198) diluted 1:400 in 10 % donor goat serum and 0.4 % Triton X-100 in PBS. The slices were then washed twice in PBS and incubated for 1.5 h at room temperature in Alexa Fluor 488 goat anti-rabbit antibody (Molecular Probes; #A-11008) diluted 1:800 in 10 % donor goat serum and 0.4 % Triton X-100 in PBS. The slices were then washed again in PBS three times. The slices were viewed, and images were acquired blind to the genotype using identical settings with Zeiss ZEN2 software on an LSM 880 laser confocal-scanning system coupled to a Zeiss inverted microscope. Images were taken using a 40 × oil immersion objective. Averages of four scans were collected for each image. The mean pixel intensity in the CA1 cell body region was determined using ImageJ 1.46r. The pixel intensity corresponding to individual CA1 neurons (identified by a threshold mask) was averaged on a per slice basis and comparisons were made between treated and untreated slices in terms of absolute fluorescence [arbitrary units (AU)], measured using identical acquisition parameters, and the % change in pixel intensity in BDNF- or forskolin-treated slices over the corresponding untreated controls was calculated. Immunofluorescence data was acquired from a total of 11 wild-type and 13 MSK1 KD mice from which between 1 and 5 slices each were used for the imaging studies for each experimental condition (control, BDNF, and forskolin). On average, approximately 70 neurons were analysed per slice across the various experimental conditions. The specificity of the phospho-CREB primary antibody was confirmed by testing the antibody in hippocampal slices prepared from a conditional CREB S133 mutant mouse in which S133 is mutated to alanine (CREB S133A; Wingate et al., 2009). No fluorescent signal was detected in CA1 pyramidal neurons (data not shown).

## Behavior

Male MSK1 KD mice (n = 12) and control littermates (n = 12), aged 15 weeks at the start of the analysis, received daily handling during the last week of a quarantine period, and were subject to a total of five weeks of experimentation. During this period, they were group housed, had *ad libitum* access to food and water, and were maintained on a light/dark cycle of 14/10 h with lights on at 8 A.M. All mice were trained and tested “blind” for genotype.

### The watermaze

The open-field watermaze is 2 m in diameter and was housed in a large laboratory room with prominent extramaze cues. The water was filled and drained daily, maintained at 25 ± 2°C, and made opaque by the addition of 500 ml of liquid latex. Each mouse was gently placed into the water facing the side walls at one of the four preplanned start positions (North, South, East, and West). If a mouse failed to reach the platform after 90 s (during training) or 60 s (probe tests) had elapsed, it was guided by hand to the escape platform. Once on the platform, each mouse was allowed to remain for 30 s. After 30 s, the mouse was taken away from the platform using a paint roller and put under a heat lamp until the next trial. The swimming path of the mice was tracked using the Actimetrics Watermaze software (Coulbourn Electronics).

### Visible cue task in the watermaze

Curtains were drawn around the pool to exclude the extramaze cues. Escape from the water was via a single moveable platform of 20 cm in diameter located 1.5 cm beneath the water surface. The platform was made visible by placing upon it a red/black plastic ball (about 20 cm in height). Mice were tested in groups of no more than six. Each mouse was given 3 d of training (four trials/day; *t* = 90 s). Each cardinal point was used once as the starting position on each day. The intertrial interval was 10 min, and the platform location was changed across trials.

### Spatial reference memory task

Curtains were absent for the rest of the spatial training, in order for mice to use extramaze cues to build up a spatial representation of the room. The escape platform, still beneath the water surface, was now uncued and remained at the same spatial location throughout the spatial reference memory (SRM) training, forcing the animals to use a spatial strategy to retrieve the platform location. The animals were subjected to 5 d of training with four trials/day, and an intertrial interval of 10 min. As in the cue task, mice were released from each cardinal point in a random order. Twenty-four hours after the last trial, a probe test (PT1) was conducted in which the platform was removed and the animal placed in the pool to swim for 60 s.

### Serial spatial memory task

The animals were then subjected to serial spatial learning, a series of five spatial reference memory tasks trained to a criterion of performance as described by Chen et al. (2000) and Daumas et al. (2008). Each task constituted a separate spatial problem, with all five problems taking place in the same watermaze in the same experimental room (starting 24-48 h after SRM completion). The platform location was varied from placement on an inner “virtual” ring (1 m in diameter) or an outer ring (1.5 m in diameter). In this way, the location differed between problems but remained the same within each day of training and each spatial problem, until the chosen criterion was reached. The animals had a maximum of 32 trials to acquire a spatial problem task, but if they reached the criterion of an average escape latency of <20 s on three consecutive trials, the training for the given spatial problem was stopped. The training for the next spatial problem began 2 d later. There was a maximum of eight trials per day, with an intertrial interval of 10 min. If an animal did not reach the platform within 90 s, it was directed to the platform using a small paint roller and allowed to rest for 30 s.

In order to assess the strength of memory for each platform location, a probe test was conducted. To study short-term memory the probe test was given 10 min after reaching criterion on each of the five problems. To study long-term memory the probe test was given 24 h after reaching criterion. A 13 cm in diameter Atlantis platform was used to avoid extinction between probe tests. This platform, which was unavailable to the mice during the test, is software-driven to rise after the 60 s of the probe trial had elapsed. The animals had 30 additional seconds to find the platform and, if they did not, were guided to the platform with a paint roller and left there for 30 s.

### Massed spatial watermaze task

A week after the end of the serial spatial task, mice were submitted to four consecutive sessions of four trials, with an intersession delay of 15 min, during which they were returned to their home cage. The Atlantis platform was submerged 0.5 cm beneath the surface of the water. Animals were introduced in the maze from different starting points and allowed to swim freely or until they reached the platform. Mice failing to find the platform within 90 s were gently guided to the platform and left on it for 30 s. The starting positions were determined in a pseudorandom order, and the sequence of starting locations was randomized such as each of them was used four times during the four training sessions.

Ten minutes, 24 h, and 7 d after the last training session mice were submitted to a probe test using the Atlantis platform, which rose after the 60 s of the probe trial had elapsed, after which mice were allowed to search for it for an additional 30 s.

## Statistical analysis

Statistical analysis was performed using IBM SPSS Statistics 22 using the tests described in Table 1. Statistical significance was set at *p* < 0.05.

**Table 1:**
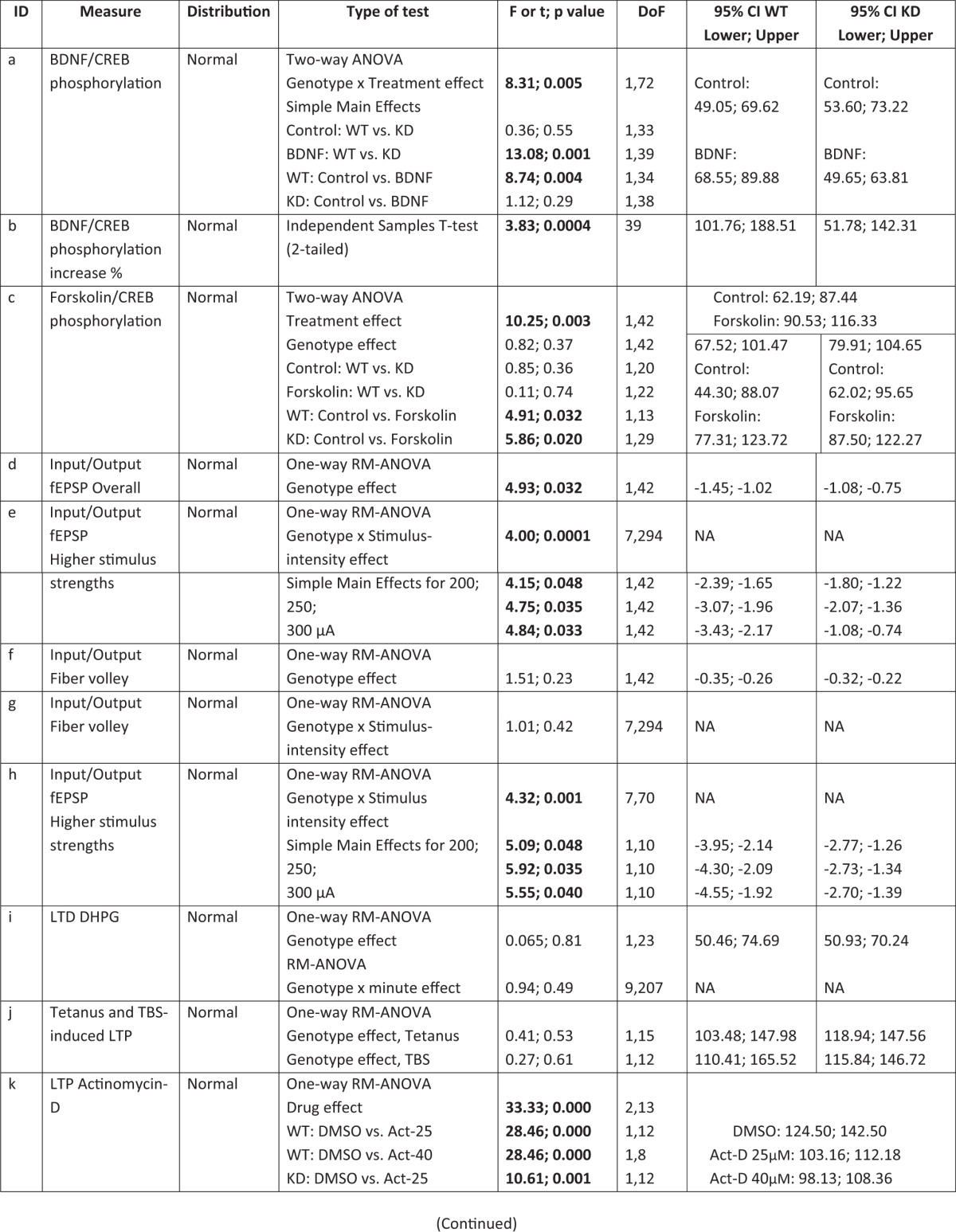

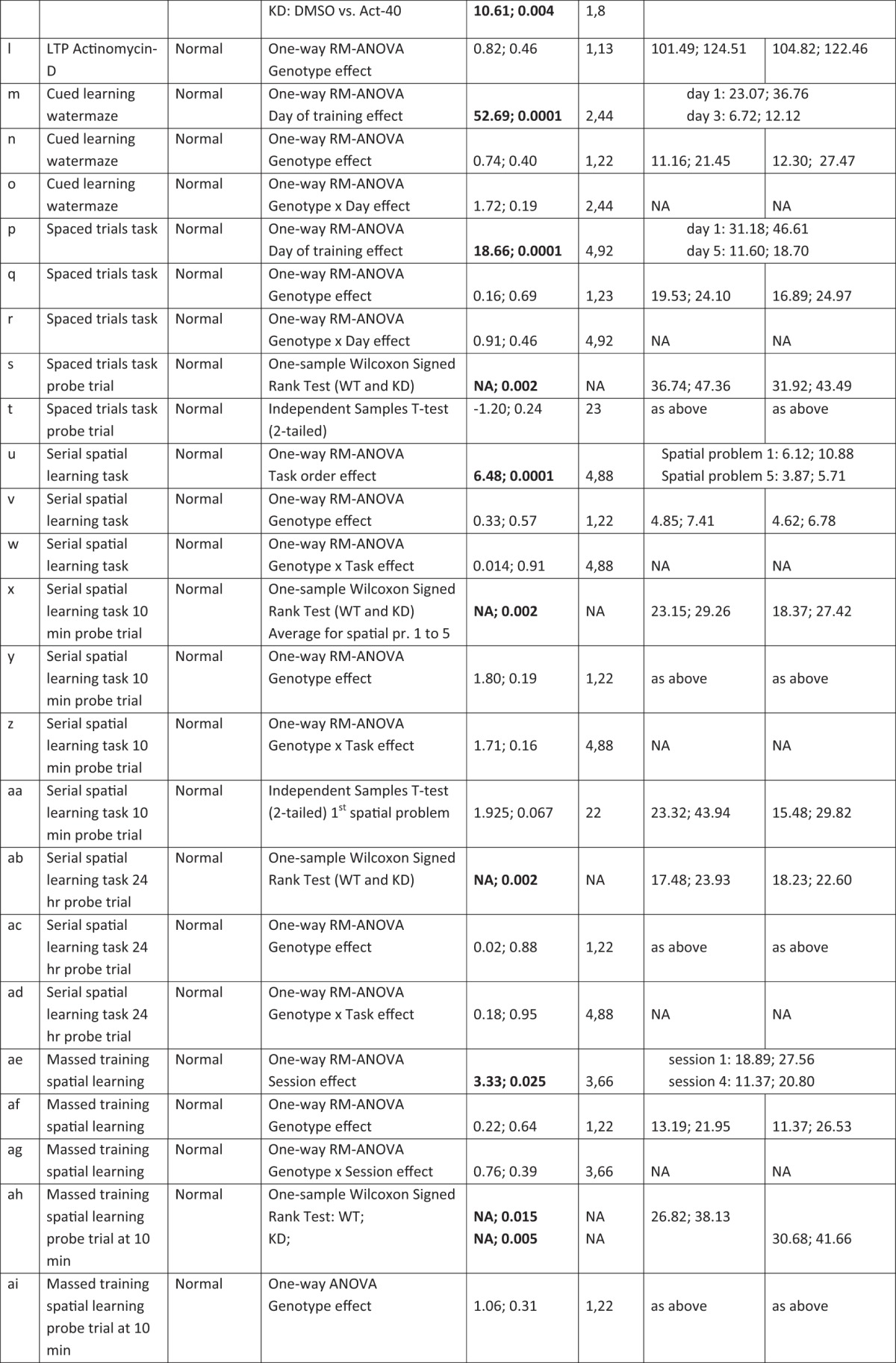
Results statistics summary

## Results

### MSK1 is required for BDNF-dependent phosphorylation of CREB S133

**Figure 1.**
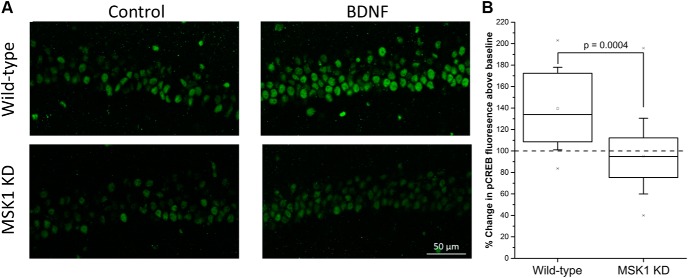
MSK1 is necessary for BDNF-dependent CREB phosphorylation in area CA1. ***A***, Treatment of wild-type hippocampal slices (upper panels) with BDNF (50 ng/ml; 10 min) resulted in a robust increase in CREB phosphorylation in CA1 neurons, compared to control untreated slices, as indicated by changes in immunofluorescence associated with a monoclonal antibody directed at the phosphorylation of CREB S133. In contrast, BDNF induced little or no effect on CREB phosphorylation in slices taken from MSK1 KD mice (lower panels). ***B***, Box and whisker plot showing mean (open square), median, 25 and 75 percentile (box), and ±1 standard deviation (whiskers) of data from 21 BDNF-treated slices from eight wild-type mice and 20 BDNF-treated slices from 10 MSK1 KD mice. The specificity of the antibody for the phosphorylation of CREB S133 in CA1 pyramidal neurons was confirmed using a mouse expressing a CREB S133-alanine point mutation (data not shown).

To confirm the kinase-dead nature of the mutation in the MSK1 gene in the MSK1 KD mutant mice, we conducted a series of experiments utilizing acutely-prepared hippocampal slices taken from wild-type and MSK1 KD mice of an age similar to those used for the electrophysiology and behavioral studies. Incubation of wild-type hippocampal slices with BDNF (50 ng/ml; 10 min) resulted in a significant increase in CA1 immunoreactivity for phosphorylated CREB at S133 (79.2 ± 5.1 AU; 21 slices from eight mice^(a)^) compared to parallel control, untreated slices from the same animal (59.3 ± 4.8 AU; 15 slices from eight mice; *p* = 0.004^(a)^; Fig. 1*A*, upper panels). There was little or no increase in CREB phosphorylation in slices taken from MSK1 KD mice (63.4 ± 4.7 vs 56.7 ± 3.4 AU in BDNF; both 20 slices from 10 mice; *p* = 0.29^(a)^; Fig. 1*A*, lower panels). There was no difference in basal pCREB fluorescence between the two genotypes (*p* = 0.55^(a)^). A comparison of the BDNF-induced change in immunofluorescence showed a significant BDNF-dependent increase in CREB phosphorylation in wild-type mice (139.5 ± 8.4%, n = 21 slices from eight mice^(b)^), but no overall change in pCREB immunoreactivity in the CA1 region of slices taken from MSK1 KD mice (95.3 ± 7.9%, n = 20 slices from 10 mice; t_39_= 3.83; *p* = 0.0004^(b)^; Fig. 1*B*). That CREB could be phosphorylated in MSK1 KD mice was demonstrated by the incubation of slices from wild-type and MSK1 KD mice in the adenylate cyclase activator forskolin (50 µM; 10 min; data not shown). Basal phospho-CREB immunofluorescence was similar across both genotypes (66.2 ± 8.9 AU for wild-type; seven slices from five mice; vs 78.8 ± 7.8 AU for MSK1 KD slices; 15 slices from six mice; *p* = 0.37^(c)^) and rose to 100.5 ± 9.8 AU and 104.9 ± 8.2 AU after forskolin application in wild-type and MSK1 KD slices, respectively (n = 8 slices from five wild-type mice and 16 slices from six MSK1 KD mice; *p* = 0.003^(c)^). Expressed as a percentage, forskolin application increased phospho-CREB immunofluorescence to a similar extent in wild-type slices (148.1 ± 12.7%; n = 8 slices from five mice) and MSK1 KD slices (137.3 ± 9.0%; n = 16 slices from six mice).

### The regulation of basal synaptic transmission, but not the probability of transmitter release, mGluR-LTD or LTP, is dependent upon the kinase activity of MSK1

To establish the role of MSK1 in synaptic function we performed electrophysiological experiments in area CA1 of hippocampal slices prepared from MSK1 KD and wild-type mice.

**Figure 2.**
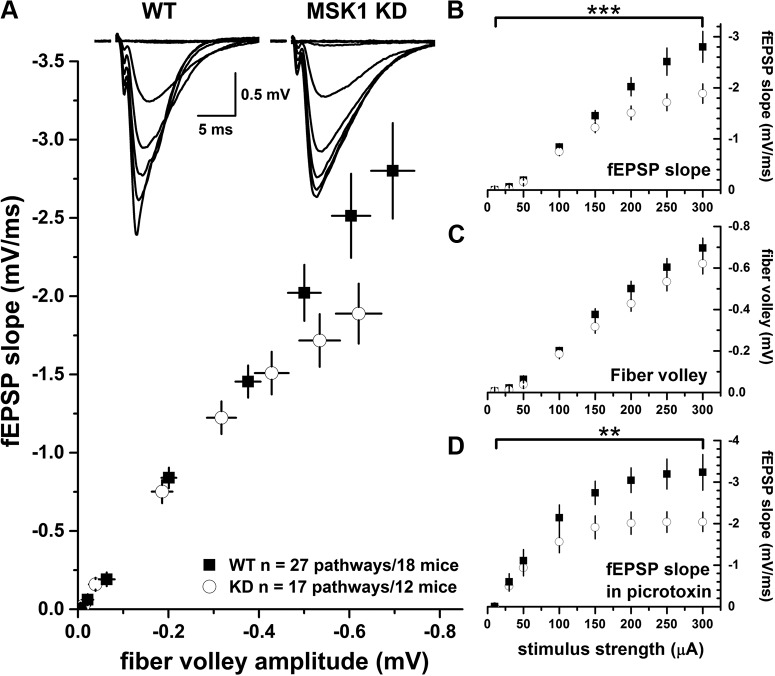
MSK1 KD mice display a deficit in basal synaptic transmission which is not due to increased GABAergic inhibition. Basal synaptic transmission was measured in the CA1 region of hippocampal slices from wild-type (black squares) or MSK1 KD (white circles) mice. ***A***, Plot of fibre volley amplitude versus fEPSP slope demonstrates that MSK1 KD mice show reduced synaptic transmission at higher stimulus strengths. Inset is representative fEPSPs at 10 to 300 µA stimulus strengths for both genotypes (n = 27 pathways from 18 wild-type mice and 17 pathways from 12 MSK1 KD mice). This deficit is also observed when fEPSP slope is plotted against stimulus strength (***B***; ***p = 0.0001; genotype x stimulus strength interaction). In contrast, a plot of fibre volley amplitude against stimulus strength (***C***) showed no significant difference between the genotypes. ***D***, The deficit in fEPSP was maintained when the experiments were repeated in the presence of 50 µM of the GABA_A_ receptor antagonist picrotoxin (n = 5 pathways from four wild-type mice and seven pathways from five MSK1 KD mice; **p = 0. 001; genotype x stimulus strength interaction). Error bars represent SEM.

The construction of stimulus input/output curves (10-300 µA) revealed a mild reduction in excitatory synaptic transmission in the MSK1 KD mutants (n = 12 mice; 17 pathways) when compared to wild-type animals (n = 18 mice; 27 pathways). This was apparent when the fEPSP slope was compared to the presynaptic fiber volley (Fig. 2*A*), or when the slope of the fEPSP was plotted as a function of the stimulus strength (Fig. 2*B*): There was a main effect of genotype (*p* = 0.032^(d)^) and a significant interaction between genotype and stimulus intensity (*p* = 0.0001^(d)^) at the higher stimulus strengths (200-300 µA; *p* = 0.048, *p* = 0.035, and *p* = 0.033, respectively^(e)^). However, there was no appreciable difference in the size of the presynaptic fiber volley between mutant and wild-type mice *p* = 0.23^(f)^) nor was there a fiber volley genotype x stimulus intensity interaction (*p* = 0.42^(g)^; Fig. 2*C*). To rule out differences in GABAergic inhibition between mutant and wild-type animals, input/output curves were constructed from slices taken from a different set of animals in the presence of the GABA_A_ receptor antagonist picrotoxin (50 µM; five pathways from four wild-type mice and seven pathways from five MSK1 KD mice). The deficit in synaptic transmission at higher stimulus strengths (200-300 µA) in slices from MSK1 KD mice persisted during antagonism of GABA_A_ receptors (*p* = 0.001) at 200, 250, and 300 µA (*p* = 0.048, *p* = 0.035, and *p* = 0.040, respectively^(h)^; Fig. 2*D*).

**Figure 3.**
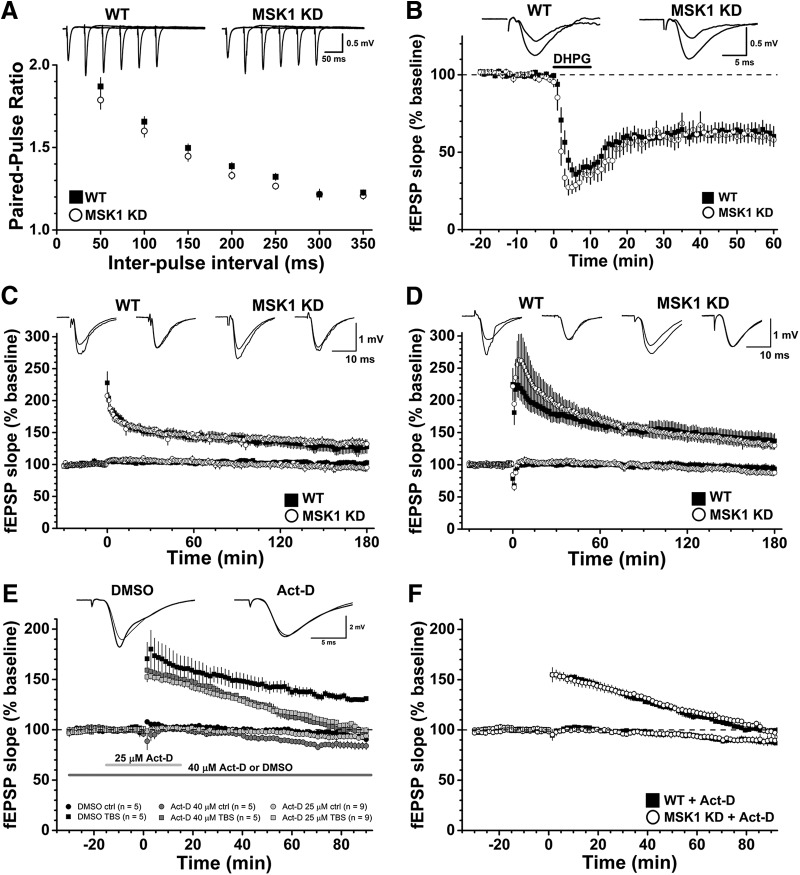
Electrophysiological characterization of MSK1 KD mice shows no deficit in paired-pulse facilitation, mGluR-LTD, or LTP. ***A***, Paired-pulse facilitation in area CA1 was measured in hippocampal slices prepared from adult wild-type (black squares; 21 pathways from 18 animals) or MSK1 KD (white circles, 22 pathways from 19 animals) mice. Representative families of paired fEPSPs taken over the 50 to 250 ms interstimulus interval range are shown in the upper panel, and quantification of the paired-pulse ratios in the lower panel. Data are expressed as mean and error bars (where visible) represent the SEM. ***B***, mGluR-LTD was induced in area CA1 of hippocampal slices from wild-type (black squares) and MSK1 KD (white circles) mice. The GI mGluR agonist DHPG (100 µM) was applied from time 0-10 min (as indicated by the black bar on the graph) in the presence of both picrotoxin (50 µM) and the NMDA receptor glycine site antagonist L689,560 (5 µM). Inset are representative fEPSPs taken 10 min before and 50 min after the end of DHPG application in wild-type and MSK1 KD slices (n = 15 and 10 slices from 11 wild-type and 8 MSK1 KD mice, respectively). Data are expressed as mean, and error bars represent the SEM. ***C***, ***D***, Tetanus- (***C***) and theta-burst stimulation-induced LTP (***D***) were measured in the area CA1 of hippocampal slices from adult mice. Inset are representative fEPSPs taken 10 min before and 120 min after induction of LTP (at time zero) in the stimulated (left) and control (right) pathways for each genotype. Data are expressed as mean, and error bars represent the SEM. For tetanus-induced LTP, data are taken from single slices from nine wild-type and eight MSK1 KD mice, while for theta-burst-induced LTP, individual slices from seven wild-type and seven MSK1 KD mice were used. ***E***, TBS-LTP is sensitive to the transcription inhibitor Act-D. Graphs show the effects of DMSO vehicle (0.08%) applied throughout the experiment (black squares and dark grey bar; n = 5; three wild-type and two MSK1 KD mice); 40 µM Act-D applied for the duration of the experiment (dark grey squares and dark grey bar; n = 5; four wild-type and one MSK1 KD mice); and 25 µM Act-D (0.05% DMSO) applied for 15 min before and after TBS (light grey squares and light grey bar; n = 9; three wild-type mice and six slices from four MSK1 KD mice). Both concentrations of Act-D affect LTP similarly, but the lower concentration and shorter duration of application of 25 µM has less of an effect on the control pathway. Data are expressed as mean, and error bars represent the SEM. ***F***, The inhibitory effects of Act-D on late TBS-LTP are independent of genotype. Data are replotted from ***E*** but with respect to genotype instead of concentration: black squares, seven slices from seven wild-type mice; open circles, seven slices from five MSK1 KD mice. Data are expressed as mean, and error bars represent the SEM.

To test whether this deficit in basal synaptic transmission had a presynaptic component, we measured the facilitation of synaptic transmission associated with delivering pairs of electrical stimuli at 50 to 350 ms intervals. Such paired-pulse facilitation is taken as an index of the initial probability of transmitter release. There was no difference in paired-pulse facilitation between mutant (22 pathways from 19 mice) and wild-type mice (21 pathways from 18 mice), with the paired-pulse facilitation profile over the 50 to 350 ms range essentially overlapping in the two genotypes (Fig. 3*A*). Thus, as far as can be determined with paired-pulse facilitation and the similar magnitudes of the presynaptic fiber volley, the deficit in basal synaptic transmission in the MSK1 KD mice is not presynaptic and instead appears likely to have a postsynaptic locus, potentially via a reduction in synaptic AMPA-type glutamate receptors.

To test whether this impairment in basal synaptic transmission reflected a LTD-type state, we examined the LTD induced by the activation of GI mGluRs. This form of LTD requires the induction of Arc/Arg3.1 (Waung et al., 2008), and a deficit in Arc/Arg3.1 regulation has been observed in MSK1 KD neurons in response to activity deprivation (Corrêa et al., 2012), which may explain the failure to induce bidirectional homeostatic plasticity in MSK1 KD mutant neurons. Accordingly, we applied the GI mGluR agonist DHPG (100 µM; 10 min) to hippocampal slices from both MSK1 KD mutant and wild-type slices and measured fEPSPs from area CA1. DHPG caused a ∼40% inhibition of the fEPSP 60 min after the onset of DHPG application that was no different between MSK1 KD and wild-type slices^(i)^ (Fig. 3*B*).

To establish instead whether LTP, which also has a requirement for Arc/Arg3.1 (Plath et al., 2006), would be affected by the MSK1 KD mutation, we performed dual-pathway LTP experiments in both mutant and wild-type slices and used both tetanus and theta-burst stimulation (TBS) paradigms as they have been reported to recruit different intracellular signaling pathways. In particular, in contrast to single tetanus LTP, TBS is reported to recruit both ERK1/2- (Winder et al., 1999), and transcription-dependent LTP (Nguyen and Kandel, 1997) and where a deficit in slices from MSK1 KD mice might be expected to be observed.

Given the difference in basal synaptic transmission between the MSK1 KD and wild-type mice (Fig. 2), and to avoid bias associated with differential postsynaptic depolarization caused by LTP-inducing high-frequency stimulation, basal fEPSP amplitude was set to 1 mV across the two genotypes. This protocol has been adopted previously with mutant mice displaying impaired basal synaptic transmission (Patterson et al., 1996). It should be noted that while such stimulus matching is important in allowing comparisons regarding relative enhancements of synaptic transmission after high-frequency stimulation, it does so only at a fixed stimulus strength. Thus, in the absence of full input-output curves before and after LTP, it is conceivable that MSK1 KD mice would still display a deficit in basal synaptic transmission after LTP when compared to wild-type mice.

Both tetanus and TBS induced robust LTP which persisted for the duration of the experiment (180 min after high-frequency stimulation). There was no obvious difference between either the initial potentiation or the potentiation at 180 min between the two genotypes or stimulation protocols (Fig. 3*C*,*D*). For wild-type mice tetanus-induced LTP measured over the last 10 min of the experiment was 127.1 ± 9.9% (n = 9), whereas after TBS, it was 137.6 ± 10.9% (both relative to the preceding baseline; n = 7); and in the MSK1 KD mutants, the corresponding values were: tetanus-induced LTP at 132.6 ± 6.4 (n = 8) and theta-burst induced LTP at 130.9 ± 6.1% (n = 7). Neither tetanus- nor TBS-induced LTP differed significantly across the genotypes (*p* = 0.53 and *p* = 0.61^(j)^, respectively). Accordingly, it would seem that the kinase activity of MSK1 is not required for LTP, at least over the time course (3 h) of the present experiments.

To confirm that the late LTP evoked required gene transcription, we performed a series of experiments using the transcription inhibitor Act-D during TBS-induced LTP (Nguyen and Kandel, 1997). In these experiments, slices were maintained in an interface chamber, in which a deficit in basal synaptic transmission was still observed in slices from MSK1 KD mice (data not shown). Due to the larger fEPSPs evoked from slices in interface chambers (Reid et al., 1988; Croning and Haddad, 1998; Bortolotto et al., 2011), basal fEPSP amplitudes in both genotypes were set at 3 mV.

When applied at 40 µM for the duration of the experiment (n = 5; four wild-type and one MSK1 KD mice), Act-D resulted in a return of LTP to baseline values within 80 min after TBS compared to interleaved DMSO control experiments (n = 5; three wild-type and two MSK1 KD mice^(k)^) where the LTP was maintained at ∼130% of control (Fig. 3*E*). However, a ∼15% decrease in the control pathway was observed with this protocol that could have accounted, at least in part, for the observed decrementing LTP. We therefore conducted an additional series of experiments where a lower concentration of Act-D (25 µM; n = 9; three wild-type mice and four MSK1 KD mice; Fig. 3*E*) was applied for 15 min before and until 15 min after TBS. This lower concentration and shorter duration of Act-D had a similar inhibitory effect on TBS-induced LTP, with a return to baseline at approximately 80 min, but with less of an influence (<10%) on the control pathway, and which was similar to that recorded in the DMSO control experiments. Since the above experiments combined slices from both wild-type and MSK1 KD slices, and showed that the effects of 25 and 40 µM Act-D on LTP were similar, we segregated the Act-D data on the basis of genotype (seven slices from seven wild-type mice and seven slices from five MSK1 KD mice). This analysis showed that TBS-induced LTP in both wild-type and MSK1 KD slices was equally sensitive to Act-D (Fig. 3*F*). These data confirm that LTP induced by TBS requires gene transcription for its maintenance^(k,l)^ (Nguyen and Kandel, 1997) in both wild-type and MSK1 KD mice and further indicate that transcription regulated by MSK1 is not required.

### The kinase activity of MSK1 is not required for spatial memory tasks in the watermaze

**Figure 4.**
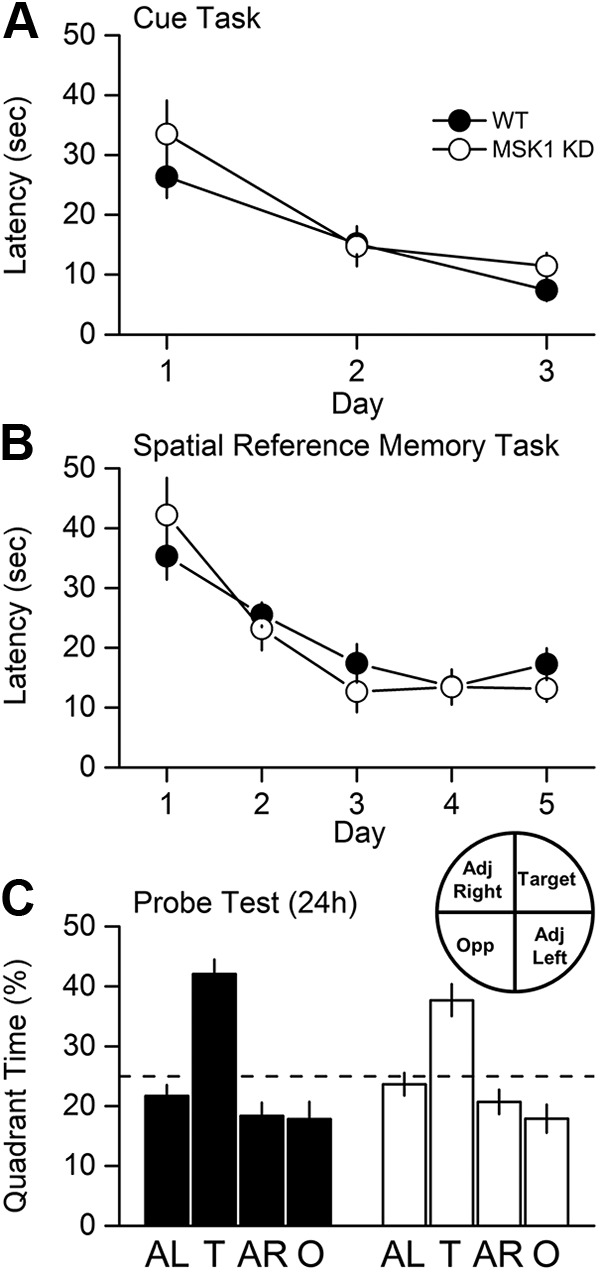
MSK1 KD mice display no deficit in the watermaze test for spatial reference memory. ***A***, The ability to locate a visible platform in a 2-m pool was assessed by a 3-d visual cue task as described in Daumas et al. (2008). Both wild-type (WT) and MSK1 KD mice improved their latency to the platform with time at an equivalent rate. ***B***, Spatial reference memory was assessed using a standard 5-d protocol (four spaced trials/day, 10-cm hidden platform, probe test after 24 h). No learning deficit was observed during training. ***C***, The retention “probe” test indicated good memory in both groups, revealed as greater time spent searching in the training quadrant. Broken line, chance level = 25 %.

**Figure 5.**
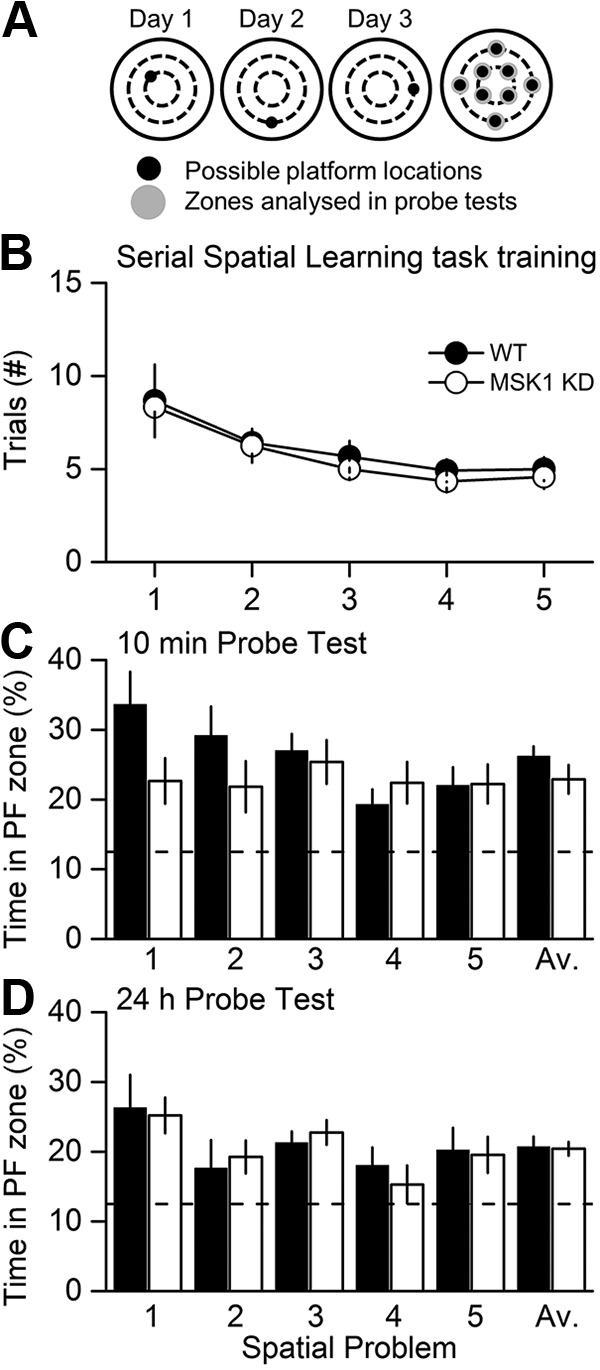
MSK1 KD mice display no deficit in the watermaze test for serial spatial learning. ***A***, A serial spatial learning task was used to assess memory flexibility as described in Daumas et al. (2008). In this task, the platform location changes between up to eight separate locations and between days, with each location trained until the animals reached a fixed criterion of performance (latency < 20 s over three trials). The measure of learning now is not latency, but the number of trials (#) required to reach criterion. ***B***, Both groups showed similar performance. After reaching criterion for each of five serial locations, memory was assessed at 10 min (***C***) and 24 h (***D***). The proportion of time spent in the relevant platform zone (extended to 20 cm in diameter) relative to the other seven possible platform locations was analysed (broken line, chance level = 12.5 %).

**Figure 6.**
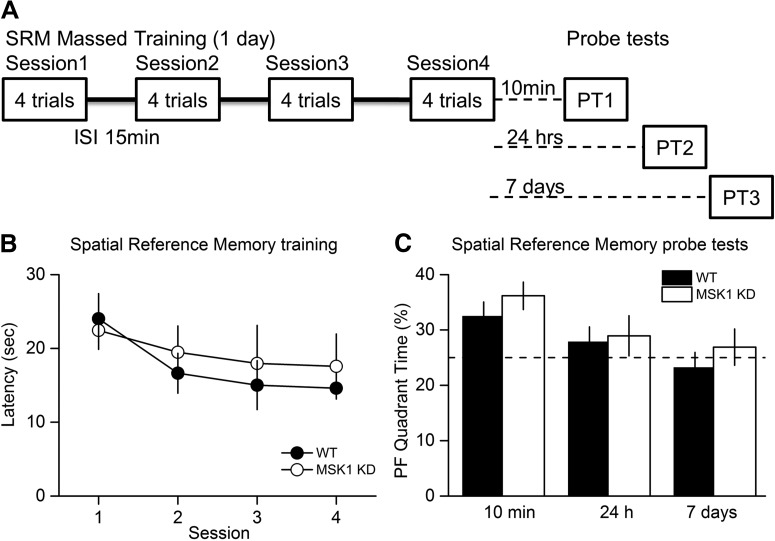
MSK1 KD mice display no deficit in the watermaze test for spatial reference memory with a massed training protocol. ***A***, In a massed training protocol (16 trials, in four trial blocks over 1 d), learning was comparable (***B***), and spatial memory assessed at 10 min, 24 h, and 7 d after training (***C***) showed the expected and more rapid forgetting over time with no group differences between WT and MSK1 KD mice.

Given previous reports that MSK1 is required for a number of learning tasks (Chwang et al., 2007; Chandramohan et al., 2008; Karelina et al., 2012), we examined MSK1 KD mice in a variety of hippocampal-dependent watermaze training protocols (Figs. 4-6). In the cued version of the watermaze (Fig. 4*A*), mice of both genotypes improved their performance over the 3 d of training with a decline in escape latency (*p* < 0.0001^(m)^); there was no significant effect due to the genotype (*p* = 0.40^(n)^), nor a significant interaction between genotype and day (*p* = 0.19^(o)^). These data indicate that the MSK1 KD mutant does not display gross motor or sensory deficits.

In the spaced trials hidden platform version of the watermaze (Fig. 4*B*), mice of both genotypes improved their performance over the 5 d of training (*p* < 0.0001^(p)^); there was no significant effect due to the genotype (*p* = 0.69^(q)^), nor a significant interaction between genotype and day (*p* = 0.46^(r)^). In a probe trial given 24 h later (Fig. 4*C*), both mutant and wild-type mice displayed selective searching in the training quadrant (42 and 38% for WT and MSK1 KD, respectively) compared to chance level (25 %; *p* = 0.002^(s)^ for both) demonstrating memory retention in both genotypes. The time spent in the training quadrant did not differ between genotypes (*p* = 0.24^(t)^).

In a further iteration of the watermaze that maximizes the opportunity for interference, mice were subjected to a serial spatial learning task in which a new platform location was used across a series of sessions (Fig. 5*A*), and the number of trials required to identify the new location of the platform taken as a measure of learning. This test proved to be exceptionally sensitive in a study of age-related changes of performance in human mutant APP mice (Chen et al., 2000). As before, the wild-type and MSK1 mutant mice performed similarly in the acquisition phase (Fig. 5*B*): a repeated-measures (RM)-ANOVA showed that mice of both genotypes improved their performance in relation to the task order presentation (*p* < 0.0001^(u)^; i.e., the number of trials required for spatial problem 1 > spatial problem 2 and 3 > spatial problem 4 and 5). It also showed that there was no effect due to the genotype (*p* = 0.57^(v)^), nor any significant interaction between spatial problem and genotype (*p* = 0.91^(w)^).

In a probe test trial given 10 min after the last training session for each platform location (Fig. 5*C*), the performance of the mutant and wild-type was similar: the average performance was above the chance level (12.5 %; *p* = 0.002^(x)^ for both WT and MSK1-KD). An RM-ANOVA showed that there was no effect due to genotype (*p* = 0.19^(y)^), nor any significant interaction between task and genotype (*p* = 0.16^(z)^). The apparent better performance by WT mice on the first spatial problem did not reach statistical significance (*p* = 0.067^(aa)^).

Similarly, mutant and wild-type mice behaved no differently from each other in a probe test given 24 h after the training sessions (Fig. 5*D*): the average performance was above the chance level (12.5 %; *p* = 0.002^(ab)^ for both WT and MSK1-KD). An RM-ANOVA showed that there was no effect due to genotype (*p* = 0.88^(ac)^), nor any significant interaction between task and genotype (*p* = 0.95^(ad)^).

Massed training tends to give rise to a weaker memory trace that fails to show lasting retention, a level of performance that may be more sensitive for revealing a subtle phenotype. To examine the decay of memory over time, both genotypes were therefore exposed to massed training in which four watermaze trials were given per session with a 15 min interval between the four sessions (Fig. 6*A*). Mice of both genotypes showed a steady decline in escape latency over the four sessions of training (*p* = 0.025^(ae)^); there was no significant effect due to the genotype (*p* = 0.64^(af)^), nor a significant interaction between genotype and day (*p* = 0.39^(ag)^; Fig. 6*B*).

Mice were then tested for memory of the location of the platform 10 min, 24 h, and 7 d later (Fig. 6*C*). At the 10 min probe test only, mice of both genotypes spent more time in the platform quadrant than the chance level (25 %; *p* = 0.015 and 0.005^(ah)^ for WT and MSK1 KD, respectively), but the percentages did not differ between genotypes (*p* = 0.31^(ai)^). Forgetting was observed in both groups over 24 h and 7 d.

These data indicate that, while there is a deficit in BDNF signalling to CREB and basal synaptic transmission in the MSK1 KD mouse, this does not translate into an impairment of mGluR-LTD, LTP or in long-term hippocampal-dependent spatial learning in the watermaze. Moreover, intact long-term learning and memory in the watermaze is consistent with intact LTP in the hippocampus in the mutant mice.

## Discussion

Our main findings are that mice harboring a knock-in kinase-dead mutation of MSK1 display a deficit in BDNF-dependent phosphorylation of CREB and of basal synaptic transmission, but not of paired-pulse facilitation, mGluR-LTD or LTP induced by either tetanic or theta-burst stimulation. In addition, MSK1 mutant mice showed no impairment in various tests of spatial memory in the watermaze. These data suggest that the kinase activity of MSK1 is not directly required for several forms of synaptic plasticity or spatial learning and memory, at least under standard housing conditions. Instead, our observations of changes in basal synaptic transmission, and thus the long-term regulation of synaptic strength, support observations of a deficit in homeostatic and experience-dependent plasticity in MSK1 KD mutants (Corrêa et al., 2012).

### MSK1 as a CREB S133 kinase

The immunofluorescence associated with the phosphorylation of CREB at S133 was similar under basal conditions between MSK1 KD and wild-type mice. This suggests that under normal circumstances the many CREB kinases, which include CaMKs, PKA, and RSKs (Flavell and Greenberg, 2008; Sakamoto et al., 2011; Kida and Serita, 2014), ensure an appropriate level of CREB S133 phosphorylation and presumably the maintenance of CREB-dependent transcription. The wide variety of CREB S133 kinases and their recruitment in an activity-dependent manner also potentially explains the minimal synaptic and behavioral phenotype of the MSK1 KD mutant mouse. Thus, there may only be specific circumstances in which MSK1 is recruited as a CREB S133 kinase, for example, in response to environmental enrichment (Corrêa et al., 2012; Karelina et al., 2012) or, interestingly, to molecules such as BDNF (Arthur et al., 2004), which is believed to play an important role in mediating the beneficial effects of enrichment on neuronal structure, synaptic function, and cognition (Cowansage et al., 2010; Bekinschtein et al., 2011).

Accordingly, stimulation of hippocampal slices with BDNF resulted in an increase in phosphorylation of CREB S133 in CA1 neurons of wild-type mice but not in MSK1 KD mice. These observations confirm the long-held view of MSK1 as a CREB kinase (Deak et al., 1998; Reyskens and Arthur, 2016) and, in particular, the observations made in primary neuronal cell culture of MSK1 and MSK2 single and double knock-outs of the necessity for MSK1 in neurotrophin-mediated phosphorylation of CREB (Arthur et al., 2004). However, as others have shown, MSK1 is also downstream of other signalling cascades that culminate in CREB phosphorylation, including that of the cAMP/PKA pathway (Frödin et al., 1994; Delghandi et al., 2005). Although we observed no obvious deficit in forskolin-stimulated CREB phosphorylation in slices from MSK1 KD mice, which may reflect a dominant contribution of PKA under our experimental conditions, others have reported that the Ca^2+^-stimulated adenylyl cyclase/PKA pathway recruits MSK1 in a subset of CA1 neurons in response to contextual fear conditioning (Sindreu et al., 2007). Since the number of CA1 neurons in our analysis of CREB phosphorylation were similar across genotypes and basal and stimulated conditions (∼70 cells/slice), we do not see evidence of subsets of responding neurons. This may reflect the bath application of BDNF and forskolin, as opposed to the pathway specificity of a behavioral stimulus.

### Role of MSK1 in synaptic transmission and plasticity

Despite there being a number of studies describing various roles for MSK1 in neuronal and cognitive function, there have been no reports to date of synaptic plasticity in MSK1 mutant mice. The observations we have made here suggest that the kinase activity of MSK1 is necessary to regulate basal synaptic transmission as MSK1 KD mice showed smaller CA1 fEPSPs in response to stimulation of the afferent Schaffer pathway, a phenotype they share with BDNF knock-out mice (Patterson et al., 1996). In the MSK1 KD mutants, this impairment was not due to reduced excitability or number of presynaptic fibres, since the amplitude of the presynaptic fibre volley was no different from wild-type animals. In addition, this deficit did not reflect either an increase in inhibitory GABAergic synaptic transmission, as the difference between mutant and wild-type slices persisted in the presence of a GABA_A_ receptor antagonist, or reduced probability of transmitter release since the paired-pulse facilitation profile was no different between mutant and wild-type slices. Instead, one possibility is that this deficit reflects reduced numbers of postsynaptic AMPA receptors, which mediate the majority of excitatory synaptic transmission at these synapses. Indeed, in whole-cell recordings from MSK1 KD and wild-type neurons, a ∼10-15 % reduction in the amplitude of miniature excitatory postsynaptic currents (mEPSCs) in hippocampal slices from animals of a similar age was observed (Corrêa et al., 2012). This observation is in contrast to the situation in cultured neurons prepared from neonatal animals where mEPSCs are larger in neurons prepared from MSK1 KD mice, likely due to the increased cell surface expression of GluA1 (but not GluA2) AMPA receptor subunits (Corrêa et al., 2012). This suggests either that the influence of MSK1 on synaptic strength may be developmentally regulated, or that homeostatic synaptic changes induced by the preparation of cultured neurons and acute brain slices do not occur in the MSK1 KD mutants.

The mechanism underlying the deficit in synaptic transmission is not clear but could potentially involve the reduced BDNF-dependent stimulation of CREB phosphorylation in hippocampal slices we observed. Such a deficit in the ERK signalling cascade could give rise to deficits in the induction of the immediate early gene Arc/Arg3.1 (Korb and Finkbeiner, 2011). Arc/Arg3.1 has been implicated in regulating synaptic strength via its association with the endocytotic proteins dynamin and endophilin (Chowdhury et al., 2006). Indeed, MSK1 KD mutants failed to downregulate Arc/Arg3.1 during prolonged (24 h) activity deprivation *in vitro*, and, likely as a consequence, the expected increase in mEPSC amplitude was not observed (Corrêa et al., 2012). Similarly, the larger spines reported in MSK1 KD neurons (Corrêa et al., 2012) has parallels with the increased number of larger spines in the Arc/Arg3.1 knock-out (Peebles et al., 2010). However, mGluR-LTD, which requires Arc/Arg3.1 for its induction (Waung et al., 2008), was not affected by the loss of MSK1 kinase activity suggesting that there are alternative means by which Arc/Arg3.1 can be regulated to support mGluR-LTD.

The LTP induction protocols used in this study evoked long-lasting (3 h) potentiation of synaptic transmission in slices from both wild-type and MSK1 KD mice. Such longevity has been previously reported for TBS-induced LTP (Nguyen and Kandel, 1997) but is not considered to be a feature of LTP induced by a single tetanus. However, it is clear from a number of studies that long-lasting LTP can be induced by a single tetanus (Bortolotto and Collingridge, 2000; Fonseca et al., 2004; Capron et al., 2006; Fonseca et al., 2006a; Fonseca et al., 2006b; Sajikumar et al., 2008; Villers et al., 2012), although there is debate as to whether or when this requires protein synthesis, as there is for TBS-induced LTP (Lynch et al., 2015). Such factors that may influence the persistence of LTP after a tetanus include the strength (Tsokas et al., 2005) or the frequency of basal synaptic transmission, with less frequent stimulation, as seen in alternating two pathway experiments such as ours, prolonging the magnitude and duration of the tetanus-induced LTP (Fonseca et al., 2006a; Villers et al., 2012).

Given the persistence of the LTP seen in our experiments, the lack of effect of the MSK1 KD mutation is at first glance somewhat surprising given that: (1) MSK1 phosphorylates CREB; (2) CREB has been implicated in synaptic plasticity, albeit with some controversy (Sakamoto et al., 2011); (3) the original CREB knock-out mouse showed a deficit in LTP over a period of 90 min (Bourtchuladze et al., 1994); (4) the MAPK/ERK pathway may be recruited preferentially by TBS (Winder et al., 1999); and (5) the ensuing LTP is sensitive to inhibition of transcription (Nguyen and Kandel, 1997).

One potential, if unlikely, explanation is that the mutation of the MSK1 gene has allowed an additional form of LTP to be evoked, in response to both TBS and tetanic stimulation, that displays identical magnitude, kinetics, and duration (to 3 h) to that evoked in slices from wild-type mice. It is thus superficially indistinguishable, to the point that it too is dependent upon transcription, at least for TBS-induced LTP. This will remain a possibility until a full biophysical and pharmacological characterization of LTP in MSK1 KD slices is performed.

A more parsimonious interpretation of the lack of an effect of the MSK1 KD mutation on LTP may arise from the work using residue-specific mutations of the BDNF TrkB receptor: an LTP deficit, over a similar time course to the present experiments (3 h), was only observed in mice with a mutation in TrkB Y816, which signals to PLCγ, CaM kinase IV, and CREB, and not mutations in TrkB Y515, which signals via Shc and the MAPK cascade, downstream of which is MSK1 (Korte et al., 2000; Minichiello et al., 2002).

### MSK1 and spatial learning

Previous reports have shown that MSK single or double knock-outs are impaired on the watermaze, fear conditioning, the Barnes maze, and in the display of behavioral immobility in the forced-swim test (Chwang et al., 2007; Chandramohan et al., 2008; Frenguelli and Corrêa, 2012; Karelina et al., 2012; Reyskens and Arthur, 2016). However, in our studies of MSK1 KD mice, we observed no deficit in various watermaze paradigms. This is consistent with the intact LTP we recorded but is at odds with previous work in other laboratories using MSK1 knock-outs. One potential explanation is that, especially in the context of the previous watermaze studies (Chwang et al., 2007), subtle differences in the way the test is conducted or the animals housed could influence the signalling pathways recruited. Indeed, other paradigms such as fear conditioning or forced swimming could recruit MSK1-dependent signalling pathways and underpin learning in those models.

An alternative explanation is that that MSK1 subserves two functions, one as a kinase and the other as a scaffolding partner in a signalling complex. The latter possibility has been made all the more likely by the observation that MSK1 forms a complex with the glucocorticoid receptor and ERK1/2 for optimal phosphorylation (serine 10) and acetylation (lysine 14; via Elk-1) of histone H3. This complex resulted in the induction of c-Fos and Egr-1 during a 15-min forced swim test (Gutierrez-Mecinas et al., 2011). Loss of the entire MSK1 protein in the knock-out may thus compromise this scaffolding role as well as the kinase pathway, and its effect may be more pronounced in tests associated with increased stress, for example, fear conditioning, forced swimming and the use of aversive motivation in the Barnes maze. Thus, whereas the MSK1 KO studies have to date been interpreted as relating to the loss of kinase activity, our use of the kinase-dead (KD) mutation raises questions about this interpretation.

## Conclusions

Mice with an inactivating kinase-dead knock-in mutation in the N-terminal kinase domain MSK1 show normal mGluR-LTD, tetanus and theta-burst LTP, and were unaffected in spatial memory tasks in the watermaze. However, these mice display a deficit in basal synaptic transmission and in BDNF-mediated phosphorylation of CREB. These data suggest that MSK1 may be important in regulating long-term, adaptive neuronal properties, rather than the acute response to experience. This is consistent with previous observations that demonstrate the necessity for MSK1 in homeostatic synaptic scaling *in vitro* and environmental enrichment-induced synaptic plasticity *in vivo* (Corrêa et al., 2012).

## Synthesis

Reviewing Editor: Pablo Castillo, Albert Einstein College of Medicine Decisions are customarily a result of the Reviewing Editor and the peer reviewers coming together and discussing their recommendations until a consensus is reached. When revisions are invited, a fact-based synthesis statement explaining their decision and outlining what is needed to prepare a revision will be listed below. The following reviewer(s) agreed to reveal their identity: Isabel Muzzio, Bryen Jordan

Your manuscript has been evaluated by two reviewers. The following is a synthesis of the online discussion. The reviewers agree that your findings showing non-kinase actions of MSK1 are interesting. Given the previous report (Corrêa et al., 2012) that characterized the kinase-dead MSK1 mutant mouse, they understand the conceptual advance of the present study is incremental, but still worth reporting as long as you properly address their concerns. A major limitation is that no clear mechanism emerges for the non-kinase functions of MSK1 (e.g. there is little to no attempt to determine how MSK1 reduces basal synaptic transmission). However, the reviewers acknowledge that addressing this important point is a major undertaken that may go beyond the scope of this study. The reviewers also raised a number of technical and conceptual concerns that should be thoroughly addressed in the revision. Chief among them are the demonstration that no MSK1 kinase activity remains in the MSK1-KD mouse and that the induction protocol used in this study triggers transcription-dependent LTP. Reviewer 2 also raised significant concerns regarding CREB phosphorylation. It is expected that the revision of your manuscript should take less than three months. Below you will find the reviewers' evaluation of your manuscript.

REVIEWER #1

The article entitled "The kinase function of MSK1 regulates BDNF signaling to CREB and basal synaptic transmission, but is not required for hippocampal long-term potentiation and spatial memory" aims at characterizing the kinase role of mitogen- and stress-activated kinase 1 (MSK1). The authors use a knock-in mouse model in which the kinase activity of MSK-1 is blocked. This manipulation prevents BDNF-induced CREB phosphorylation. At the cellular level, the mutants show a mild phenotype in basal synaptic transmission. However, the mutation does not produce changes in LTP or spatial memory.

Main weakness: The novelty of this paper is limited by the fact that a previous study using the same mouse model already characterized a role for the kinase activity of MSK1 (Correa et al., 2012). The present study extends these findings by showing subtle alterations in basal synaptic transmission as well as negative results on a few forms of plasticity and spatial learning tasks. Unfortunately, without further experiments and/or manipulations these findings do not provide any important additional insight about the kinase function of MSK1. Furthermore, the authors suggest that the molecular complex involving MSK1, ERK1/2, and glucocorticoid receptors could be responsible for the previously reported learning and memory phenotypes observed in MSK1 general knockouts. However, this possibility is not directly addressed, which significantly narrows the contribution of this study.

Another problem is that it is not clear what the authors mean by the possible "structural" role of MSK1. A previous study showed that activated glucocorticoid receptors enhance ERK1/2 activity leading to MSK1 phosphorylation, which in turn produces Histone 3 acetylation. However, in the current manuscript acetylation of Histone 3 is not even mentioned. Taking into account that one of the main conclusions of this study is that the previously reported deficits in learning and memory might have been produced by this alternative signaling cascade, it would be critical to provide more information about this. In this regard, it would also be important to explain why a glucocorticoid complex that is selectively activated by stress should play a role in forms of learning and memory that do not involve anxiety or fear, such as the Barnes maze or object recognition (Karelina et al., 2012).

The electrophysiological analysis of this paper is quite limited. The authors only investigate two forms of plasticity. Furthermore, the authors claim that one 100 Hz tetanus was given to induce LTP (page 8). Unless this is a Method typo, these results do not correlate with the typical pattern observed with one train LTP. Many studies have shown that this stimulation only produces potentiation for 1 hr (short-term) rather than the sustained enhancement observed in Figure 3B.

The conclusions of the paper should not be overstated. The negative results only indicate that the kinase activity of MSK1 is not necessary for one train and theta burst LTP. Similarly, the authors should limit their conclusions to the behavioral tasks tested.

In sum, given the fact that this mutation has been previously characterized, the current data do not significantly advance our understanding of the kinase role of MSK1.

Stats

1) In Figure 5C, the authors perform analysis over the average performance across days and then run an independent t-test on each spatial problem. Each spatial task may influence how animals learn on sequential sessions. Therefore, the most appropriate analysis should be a repeated-measures ANOVA, with time and genotype as independent variables. It seems that there is a difference on the first spatial task but this comes out as non-significant using a post-hoc independent t-test (p=0.067). It would be important to corroborate this result with the appropriate statistics.

2) It is very commendable that the authors report power in a Table. However, some values seem pretty low. This point should be addressed and clarified.

REVIEWER #2

The authors characterized a transgenic mouse expressing a kinase-dead MSK1 mutant and found effects on hippocampal basal synaptic transmission, but no effects on long-term potentiation or behavioral paradigms testing learning and memory. These findings are surprising because previous work showed that MSK1 knockout mouse have impaired CREB function and rodent behavior.

This work is well done and interesting, as it suggests that the role of MSK1 in learning and memory is unrelated to its kinase function. The electrophysiological and behavioral studies are thorough and well analyzed, and therefore results are convincing. However, there are some technical and conceptual concerns that need to be addressed prior to publication.

MAJOR

1- The imaging analysis of CREB phosphorylation requires some clarification:

a) It is not clear how/why background was subtracted, but commonly used algorithms can result in image arithmetics, therefore altering intensity values. Needless to say, this in unacceptable.

b) Not clear if imaging analysis was done blind.

c) BDNF stimulation seems to upregulate the number of neurons expressing phospho CREB (in both WT and KD), more so than increasing phospho CREB in each neuron. This should be checked. A previous important but unreferenced paper (Sindreu CB, Neuron, 2007), suggests that the effects of MSK1 are restricted to a subset of CA1 neurons.

d) Control and BDNF stimulated fluorescence should be reported (not just the ratio), as it seems there is less phospho CREB in unstimulated slices from KD mice. This may address effects on synaptic scaling observed in Correa 2012.

e) Omitting the primary antibody will not test its specificity. As this can only be tested using CREB transgenics (difficult), the results of Figure 1 should be confirmed using Western blots.

2- The recording duration of LTP experiments (Fig. 3) is on the border of transcription-dependent effects. Classic experiments by Kandel, Frey and Krug in the mid 90s show that transcriptional inhibition affects LTP maintenance only 2-4 hours post induction. Moreover, the 3X, 20 second interval, 100hz stimulation protocol may not be ideal for L-LTP. While such parameters can vary significantly between labs, the authors should at least show that they are recording transcription-dependent LTP (using actinomycin D), to strengthen claims that MSK1 kinase activity does not regulate this form of synaptic plasticity.

3- This manuscript seems too biased towards MSK1, as there are no references to the historically and functionally important roles of CaMKIV, PKA, PKC and RSK2 in CREB phosphorylation at Ser-133. After all, their results show that loss of MSK1 activity has no effect on LTP and behavioral paradigms of learning and memory.

4- That the MSK1 mutation completely abolishes kinase activity is fundamental for the conclusions of this paper. The only evidence provided is a reference to Correa 2012 which describes an IP kinase assay in fibroblasts. Ideally the authors should check the phosphorylation of other MSK1 substrates in brains of MSK1 KD mice.

MINOR

1- “ an MSK1 mutant has been generated in which the kinase activity of MSK1 is selectively inactivated, but one in which protein levels of MSK1 remain close to wild-type levels (Correa et al., 2012)” does not seem accurate. An eyeball estimation suggests KD mice have >20% of total MSK1 compared to WT (Fig. 1C).

2- Was there a rationale for using only males in behavioral assays?

3- The lack of an effect on LTP is further surprising given the loss of Arc regulation shown in Correa 2012. The authors may want to look at other forms of Arc-dependent plasticity (like mGluR-LTD).
